# Signs of Mood and Anxiety Disorders in Chimpanzees

**DOI:** 10.1371/journal.pone.0019855

**Published:** 2011-06-16

**Authors:** Hope R. Ferdowsian, Debra L. Durham, Charles Kimwele, Godelieve Kranendonk, Emily Otali, Timothy Akugizibwe, J. B. Mulcahy, Lilly Ajarova, Cassie Meré Johnson

**Affiliations:** 1 Physicians Committee for Responsible Medicine, Washington, D.C., United States of America; 2 Department of Medicine, The George Washington University, Washington, D.C., United States of America; 3 Department of Veterinary Anatomy and Physiology, University of Nairobi–Kenya, Nairobi, Kenya; 4 AAP Sanctuary for Exotic Animals, Almere, The Netherlands; 5 Kibale Chimpanzee Project, Makerere University Biological Field Station, Kibale, Uganda; 6 Uganda Program, Wildlife Conservation Society, Kampala, Uganda; 7 Chimpanzee Sanctuary Northwest, Cle Elum, Washington, United States of America; 8 Chimpanzee Sanctuary and Wildlife Conservation Trust, Entebbe, Uganda; VIB & Katholieke Universiteit Leuven, Belgium

## Abstract

**Background:**

In humans, traumatic experiences are sometimes followed by psychiatric disorders. In chimpanzees, studies have demonstrated an association between traumatic events and the emergence of behavioral disturbances resembling posttraumatic stress disorder (PTSD) and depression. We addressed the following central question: Do chimpanzees develop posttraumatic symptoms, in the form of abnormal behaviors, which cluster into syndromes similar to those described in human mood and anxiety disorders?

**Methodology/Principal Findings:**

In phase 1 of this study, we accessed case reports of chimpanzees who had been reportedly subjected to traumatic events, such as maternal separation, social isolation, experimentation, or similar experiences. We applied and tested DSM-IV criteria for PTSD and major depression to published case reports of 20 chimpanzees identified through PrimateLit. Additionally, using the DSM-IV criteria and ethograms as guides, we developed behaviorally anchored alternative criteria that were applied to the case reports. A small number of chimpanzees in the case studies met DSM-IV criteria for PTSD and depression. Measures of inter-rater reliability, including Fleiss' kappa and percentage agreement, were higher with use of the alternative criteria for PTSD and depression. In phase 2, the alternative criteria were applied to chimpanzees living in wild sites in Africa (n = 196) and chimpanzees living in sanctuaries with prior histories of experimentation, orphanage, illegal seizure, or violent human conflict (n = 168). In phase 2, 58% of chimpanzees living in sanctuaries met the set of alternative criteria for depression, compared with 3% of chimpanzees in the wild (p = 0.04), and 44% of chimpanzees in sanctuaries met the set of alternative criteria for PTSD, compared with 0.5% of chimpanzees in the wild (p = 0.04).

**Conclusions/Significance:**

Chimpanzees display behavioral clusters similar to PTSD and depression in their key diagnostic criteria, underscoring the importance of ethical considerations regarding the use of chimpanzees in experimentation and other captive settings.

## Introduction

Since nonhuman animals, including chimpanzees, are frequently used in research, there is an ethical imperative to understand the potentially adverse effects of captivity and their use in research. The association of pathological behaviors with captivity in nonhuman primates has been noted for decades [1]–[9]. In fact, the relationships between captivity and adverse physical, social and psychological effects have been the foundation for many attempts to develop “models” of human psychopathology, especially following from the early work of Harry Harlow [10]. For example, it is widely recognized that premature separation from mothers reliably leads to a range of adverse behavioral and social effects in chimpanzee infants [11], [12]. Likewise, other unfavorable rearing conditions, social isolation, prolonged captivity, sensory deprivation, and use in laboratory experimentation have been reported to be contributors to behavioral pathology in nonhuman primates [13]–[16]. Several authors have demonstrated the prevalence of abnormal behaviors, ranging from whole-body stereotypies to self-injurious behaviors, in chimpanzees and other nonhuman primates in captivity [6], [15], [17], [18].

In humans, anxiety disorders, such as posttraumatic stress disorder (PTSD), and mood disorders, such as major depressive disorder, are commonly diagnosed after acute, repeated, or chronic trauma. These types of stressors can sometimes overwhelm normal physiological responses, which can cause persistent physiological and structural changes [19], [20].

Brain structures and neuroendocrine mechanisms associated with mood and anxiety disorders are shared across a wide range of vertebrates [19]–[24]. An evolutionary psychiatry framework, as described by Brüne [25], Stevens and Price [26], Fabrega [27], and others, also predicts similarities across species in genetic, developmental, and environmental risk and protective factors for psychopathology. Similarly, changes, disruption, or dysfunction of common neuropsychiatric systems can result in similar patterns of symptom expression.

The hippocampus, found in all mammals, is a brain structure involved in memory storage and retrieval. In humans, PTSD has been associated with reductions in hippocampal volume or activity, perhaps because of recurrently and chronically elevated levels of cortisol, followed by down-regulation of the hypothalamic-pituitary-adrenal axis. Abnormalities of this axis have been described in animals subjected to confinement, restraint, isolation, or surgical procedures [22], [23].

Qualities exhibited by chimpanzees demonstrate that they have perceptual abilities, memory, cognition, and emotions, all of which have varying levels of importance for the development of psychiatric disorders. Like human children, young chimpanzees inspect and manipulate objects that do not function as expected in a familiar task, reflecting a foundation of causative understanding [28]. When adequate information about causation is apparent in problem-solving tests, young, wild-born chimpanzees ignore irrelevant information and improvise solutions [29]. Chimpanzees' understanding of causal relationships in the environment is further illustrated by varied, sophisticated use of tools, and dozens of tool use have been described in wild chimpanzee adults [4], [30], [31].

Chimpanzees demonstrate self-awareness in standard mirror tests [32], even in infancy [33]. As adults, chimpanzees have demonstrated joint attention (gaze following with a similar focus of attention) [34], selective cooperation [29], and deception [35], each reflecting an awareness of what others know. Learning and memory develop rapidly during the first 2 years of life [36], and young chimpanzees can outperform adult humans on complex short-term memory tests [37]. Moreover, chimpanzees are capable of remembering objects [38] and place [4] following intervals of years or even decades since prior exposure.

Despite the many similarities between humans and nonhuman primates, it is uncommon to study psychopathology in nonhuman primates using the terms and tools of human psychiatry. Moreover, pathological behaviors in these individuals are often described in isolation without being distilled into recognizable syndromes. Still, such behaviors are widely recognized as abnormal [39], in that they are not typically present in wild populations [4], [40].

There is empirical support for using psychiatric diagnostic criteria for nonhuman primates. Complex PTSD, depression, and other psychiatric conditions have been diagnosed in other species, including chimpanzees [14], [22], [23], [41]–[46], although it is unclear how appropriate the DSM-IV (Diagnostic and Statistical Manual of Mental Disorders, 4^th^ Ed.) criteria [47] or other traditional methods of assessing psychopathology are for use in species other than humans.

Efforts to estimate the prevalence of psychiatric disorders in chimpanzees and other animals encounter special challenges. Diagnostic criteria for mood and anxiety disorders typically require verbal descriptions from subjects of their experiences and internal states. The inability of nonhuman primates to report symptoms presents obstacles similar to those in pediatrics, psychiatry, and geriatrics, often requiring special investigative methods, including gathering information from relevant third parties [48]–[52]. As in the care of humans, the reports of specialized observers, such as technicians and staff, have been used successfully to assess and study well-being [53]–[55] and personality in chimpanzees [56]–[59] and other primates [2] for decades.

Here, we describe an investigation to apply formal diagnostic criteria for mood and anxiety disorders to chimpanzees in which we address two fundamental questions: (a) Do traumatized chimpanzees develop posttraumatic symptoms expressed in the form of psychiatric disorders? (b) If they do, are DSM-IV diagnostic criteria for these disorders adequate to describe posttraumatic disorders in chimpanzees?

In the first part of the study, we hypothesized that behavioral disorders in chimpanzees would fit one of three mutually exclusive possibilities: (a) a number of traumatized chimpanzees would reliably meet DSM-IV criteria for PTSD or depression; (b) traumatized chimpanzees would manifest few or only transient symptoms; (c) traumatized chimpanzees would develop many of the symptoms of psychiatric disorders, but would not reliably meet DSM-IV criteria. If hypothesis (a) was supported, we would conclude that PTSD and depression occur in chimpanzees in forms similar to those described in human adults. If hypothesis (b) was supported, we would conclude that the disorders of interest do not occur in chimpanzees. Finally, if hypothesis (c) was supported by our findings, we would conclude that disorders similar to those occurring in humans occur in chimpanzees, but that the diagnostic criteria and perhaps the definitions of syndromes need revising to be appropriate for this species. For the second part of the study, we hypothesized that there would be differences between the wild and sanctuary populations in the prevalence of constellations (clusters) of abnormal behavior.

## Methods

### Ethics statement

The presented study strictly adhered to the legal requirements of the countries in which it was conducted. Approval for the study was provided by the governing body of each site participating in the study and by the Uganda National Council for Science and Technology, the Uganda Wildlife Authority, and the National Council of Science and Technology under the Ministry of Higher Education, Science and Technology in the Republic of Kenya. Exemption for institutional review board approval was determined by an independent, external institutional review board because the study did not involve research on human subjects. The study relied on long-term experience of the people who were the most familiar with individual chimpanzees and did not directly impact the chimpanzees – no pain or suffering was inflicted, and authors relied on the historical observations of people who knew the chimpanzees. Chimpanzees involved lived in the wild or in sanctuaries as close to chimpanzees' natural habitat as possible.

### Methods overview

In this study, while addressing the fundamental questions described above, we also aimed to (a) understand the nature and epidemiology of psychiatric conditions among great apes, and (b) quantify the prevalence among chimpanzees of behavior clusters that are comparable to human psychiatric disorders. We followed methods similar to those reported by Scheeringa [49] for the development of alternative criteria for PTSD in nonverbal infants and children. The study was conducted in two phases. In phase 1, we applied the DSM-IV diagnostic criteria to published case reports of traumatized chimpanzees, and we developed and tested alternative criteria for these disorders to compare them to the DSM-IV criteria. The alternative criteria developed in phase 1 were used in phase 2. In phase 2, we collected and compared observations of captive and wild chimpanzees. We applied the alternative diagnostic criteria to two groups: the first was a sample of chimpanzees currently living in sanctuaries as a result of previous use in laboratory research, illegal trade and seizure, or being orphaned; the second was a sample of chimpanzees living in the wild.

#### Phase 1

The case reports for phase 1 were published in previous scientific reports; to obtain them, we searched “PrimateLit,” a database funded by the National Institutes of Health of all primate literature from 1940 to the present (our search was completed September 30, 2009). We did a global search using the umbrella term “nonadaptive,” which includes stereotypies and other behavioral and psychological pathologies, and limited our search to the genus *Pan*. The search returned 384 results that met our initial criteria.

Articles were screened to exclude conference abstracts, books, and formats other than journal articles (240 items). After 9 items were removed because they were not in English, the remaining 135 articles were screened to determine if they met the following criteria:

1. They reported original data on one or more individually identifiable chimpanzees (i.e., they were not group-level comparisons, population proportions, etc.).
2. They reported in detail on behaviors that are recognized as pathological in animals (e.g. stereotypic locomotion, hair plucking, self-mutilation) and humans.
3. Information in the article or title suggested that there had been some potentially adverse or traumatic experience such as maternal separation, being orphaned, chronic close confinement, experimentation, social isolation, or a similar experience.

Using these inclusion criteria, we found that 20 cases in a total of 12 published papers provided adequate detail for review. Most of these articles included details for more than one individual and, in certain cases, more than one article was about the same individual(s) at different times or with a focus on different details. Information about the individual case studies and the citations for the associated articles are listed in Table 1 [14], [60]–[70].

**Table 1:** Case studies used in phase 1, sex, citation(s), and general histories.

Name	Sex	Citation(s)	General History
DP2 male aka “The Lump”	M	Turner et al., 1969	Experimental
DP1 male	M	Turner et al., 1969	Experimental
DP1 female	F	Turner et al., 1969	Experimental
186	F	Menzel et al., 1963	Experimental
188	F	Menzel et al., 1963	Experimental
Ubar	M	Clark et al., 1982	Experimental, zoo
Lulu	F	Clark et al., 1982	Experimental, zoo
Cindy	F	Clark et al., 1982	Hand reared, zoo
Brenda	F	Clark et al., 1982	Hand reared, zoo
Jeannie	F	Bradshaw et al., 2008	Experimental, sanctuary
Rachel	F	Bradshaw et al., 2008	Experimental, sanctuary
Billy Jo	M	Bradshaw et al., 2009	Experimental, sanctuary
Newt	M	Bourgeois et al., 2007	Experimental
NoName	M	Howell et al., 1997; Struck et al., 2007	Experimental
Rosalyn	F	Struthers et al., 1990	Experimental
Copper	M	Pfeiffer et al., 1978; Noon, 1991	Experimental, zoo, sanctuary
Nolan	M	Pfeiffer et al., 1978; Noon, 1991	Experimental, zoo, sanctuary
Janet	F	Pfeiffer et al., 1978; Noon, 1991	Experimental, zoo, sanctuary
Larry	M	Pfeiffer et al., 1978; Noon, 1991	Experimental, zoo, sanctuary
KB	M	Hasegawa et al., 1988	Wild, mother reared, orphaned

The 21 items in the DSM-IV comprising the criteria for PTSD and the 13 items in the DSM-IV comprising the criteria for major depression were applied to the 20 published cases. Three raters who were aware of the purpose of the study rated all 20 cases independently. Each of the raters had a background in primatology and was familiar with normative and abnormal chimpanzee behaviors. Inter-rater reliability was assessed by calculating Fleiss' kappa (Κ) and the percentage agreement for each item.

In consultation with experts in primatology, ethology, psychology, and psychiatry, we then constructed an expanded checklist of symptoms designed to account for chimpanzee/human differences. Based on careful review of inventories of chimpanzee behavior (“ethograms”) from wild, sanctuary and zoo settings, we matched behaviors previously documented among chimpanzees in those settings to specific DSM-IV diagnostic criteria for PTSD and depression. Appropriate changes to the wording of questions in the DSM-IV were also made. Our goal was to make the expanded checklist of symptoms, and therefore the alternative criteria, behaviorally anchored. We paid special attention to ensure that the clusters of symptoms (i.e. the sets of alternative criteria), as revised, had face validity, in that they were similar to the criteria for PTSD and depression listed in the DSM-IV. We asked the raters to apply the expanded checklist of symptoms to each case study. The frequency with which items were reported in the case studies, the reliability coefficients, face validity, and consultation with experts were all used in a selection process to determine whether to keep, eliminate, or modify items, in order to create a set of alternative criteria for PTSD and depression for use in the remainder of the study (Tables 2 and 3).

**Table 2:** Comparison of two sets of criteria for post-traumatic stress disorder: DSM-IV (Phase 1) and the alternative set for chimpanzees (Phases 1 and 2).

DSM-IV Criteria		Alternative Criteria	
A. The person has been exposed to a traumatic event in which both of the following were present:	(1.) The person experienced, witnessed, or was confronted with an event or events that involved actual or threatened death or serious injury, or a threat to the physical integrity of self or others.	A.	(1.) Same.
	(2.) The person's response involved intense fear, helplessness, or horror.		(2.) Deleted.
B. The traumatic event is persistently reexperienced in at least one of these ways:	(1.) Recurrent and intrusive distressing recollections of the event, including images, thoughts or perceptions.	B. Reexperiencing. 1 item needed:	(1.) Deleted.
	(2.) Recurrent distressing dreams of the event.		(2.) Deleted.
	(3.) Acting or feeling as if the traumatic event were recurring (includes a sense of reliving the experience, illusions, hallucinations, and dissociative flashback episodes, including those that occur on awakening and when intoxicated).		(3.) Deleted.
	(4.) Intense psychological distress at exposure to internal or external cues that symbolize or resemble an aspect of the traumatic event.		(4.) Emotionally upset by reminders of negative or traumatic events in the past.
	(5.) Physiological reactivity on exposure to external cues that symbolize or resemble an aspect of the traumatic event.		(5.) A physical reaction to reminders of negative or traumatic events in the past (e.g. goose bumps, heavy or irregular breathing).
C. Persistent avoidance of stimuli associated with the trauma and numbing of general responsiveness, as indicated by at least three of the following:	(1.) Efforts to avoid thoughts, feelings or conversations associated with the trauma.	C. Avoidance. 3 items needed:	(1.) Deleted.
	(2.) Efforts to avoid activities, places, or people that arouse recollections of the trauma.		(2.) Avoidance of certain activities, places or types of places, or certain individuals or groups (human or chimpanzee) that may arouse recollections of trauma.
	(3.) Inability to recall an important aspect of the trauma.		(3.) Deleted.
	(4.) Markedly diminished interest or participation in significant activities.		(4.) Lack of interest in play, food, other individuals, or grooming.
	(5.) Feeling of detachment or estrangement from others.		(5.) Social withdrawal.
	(6.) Restricted range of affect.		(6.) Less variability in facial expressions compared with other chimpanzees.
	(7.) Sense of foreshortened future.		(7.) Deleted.
D. Persistent symptoms of increased arousal, as indicated by at least two of the following	(1.) Difficulty falling or staying asleep.	D. Increased arousal. 2 items needed:	(1.) Awake or easily awakened during evening observations, difficulty falling asleep, or excessive sleep.
	(2.) Irritability or outbursts of anger.		(2.) Excessive outbursts, or easily irritated or angered.
	(3.) Difficulty concentrating.		(3.) Poor attention to tasks or difficulty concentrating.
	(4.) Hypervigilance.		(4.) Extremely watchful or “on guard”.
	(5.) Exaggerated startle response.		(5.) Easily startled or jumpy.
E. Duration of symptoms is more than 1 month.		E. Duration of symptoms reported in text using population-based trends.	
F. The disturbance causes clinically significant distress or impairment in social, occupational, or other important areas of functioning.		F. Deleted.	

**Table 3:** Comparison of two sets of criteria for major depression: DSM-IV (Phase 1) and the alternative set for chimpanzees (Phases 1 and 2).

DSM-IV Criteria		Alternative Criteria	
A. Five or more of the following, including at least (1.) or (2.):		A. Five or more of the following, including at least (1.) or (2.):	
	(1.) Depressed most of the day, nearly every day, as indicated by either subjective report (e.g., feels sad or empty) or observation made by others (e.g., appears tearful). Note: In children and adolescents, can be irritable mood.		(1.) Depressed hunched posture, social withdrawal, or easily irritated or angered.
	(2.) Markedly diminished interest or pleasure in all, or almost all, activities most of the day, nearly every day (as indicated by either subjective account or observation made by others).		(2.) Loss of interest in food, play, other individuals, or grooming.
	(3.) Significant weight loss when not dieting or weight gain (e.g., a change of more than 5% body weight in a month), or decrease or increase in appetite nearly every day. Note: In children, consider failure to make expected weight gains.		(3.) Unexpected weight loss, failure to gain weight, hoarding or gorging food, or unexpected weight gain.
	(4.) Insomnia or hypersomnia nearly every day		(4.) Awake or easily awakened during evening observations, difficulty falling asleep, or excessive sleep.
	(5.) Psychomotor agitation or retardation nearly every day (observable by others, not merely subjective feelings or restlessness or being slowed down).		(5.) Restlessness or slow or sluggishness.
	(6.) Fatigue or loss of energy nearly every day.		(6.) Deleted.
	(7.) Feelings of worthlessness or excessive or inappropriate guilt (which may be delusional) nearly every day (not merely self-reproach or guilt about being sick).		(7.) Deleted.
	(8.) Diminished ability to think or concentrate, or indecisiveness, nearly every day (either by subjective account or as observed by others).		(8.) Poor attention to tasks or difficulty concentrating.
	(9.) Recurrent thoughts of death (not just fear of dying), recurrent suicidal ideation without a specific plan, or a suicide attempt or a specific plan for committing suicide.		(9.) Deleted.
B. The symptoms do not meet criteria for a Mixed Episode.		B. Deleted.	
C. The symptoms cause clinically significant distress or impairment in social, occupational, or other important areas of functioning.		C. Deleted.	
D. The symptoms are not due to the direct physiological effects of a substance (e.g., a drug of abuse, a medication) or a general medical condition (e.g., hypothyroidism).		D. Same.	
E. The symptoms are not better accounted for by Bereavement.		E. Deleted.	

#### Phase 2

In phase 2, the expanded symptom checklist, followed by the alternative diagnostic criteria for PTSD and depression developed in phase 1, were applied to chimpanzees at eight sites (three wild sites, [n = 196] and five sanctuary sites [n = 168], including two African sanctuary sites and three sanctuary sites in North America, Asia, and Europe that housed chimpanzees formerly used in laboratory research). The five sanctuary sites were combined for the purposes of this study. The study included heterogeneous populations of various ages and both sexes: 56% of the chimpanzees living in the wild were female and 49% of chimpanzees living in sanctuaries were female. Chimpanzees in African sanctuaries had histories that included illegal trade, being orphaned, or violent human conflict. Chimpanzees previously used in laboratory research had been used in experimentation for HIV, hepatitis, or other purposes. These chimpanzees had been exposed to various degrees and periods of sustained confinement, disruption of primary attachments, removal from social groups, repeated phlebotomy, capture, anesthetic injections via syringe or dart guns, invasive procedures, induced diseases, or chronic isolation. A small sample of the chimpanzees living in the wild had histories of injuries resulting from snares, spears, or similar objects.

Using the expanded checklist of symptoms developed in phase 1, caregivers at the sanctuaries provided information about chimpanzees that was based on their past observations of the chimpanzees' persistent behaviors during the time in which the raters knew these individuals. We used parallel methods to assess wild chimpanzees. Trained individuals who work at selected field sites with chimpanzees were asked to complete the surveys for specific individuals. In each of the eight settings, three surveys were completed by three different observers for each chimpanzee. While respondents were informed that the study related to primate behavior, all were masked to the study hypotheses. There was some variation in job duties among respondents, e.g. night versus daytime rounds, which provided opportunities for observations in different contexts. Responses were made independently and without consultation. This approach capitalized on the intimate knowledge and long-term experience of the people who were the most familiar with individual chimpanzees while minimizing the impact on the chimpanzees. Existing historical behavioral data from chimpanzees at these field sites provided further context for corroborating findings in any cases that proved particularly unusual or informative. Test-retest was assessed in a random sub-sample of raters and chimpanzees at each site. This random subset of raters was asked to complete the questionnaire twice, separated by an approximately 2-week interval. Intra-rater percentage agreement was used to determine the reliability of the raters' assessments. Cohen's kappa (K) was calculated to produce the most conservative estimate of intra-rater reliability.

Additionally, medical and behavioral records and other source documents were reviewed for each chimpanzee, with special attention paid to previous exposures to injury, illness, and related information. Other demographic and life history data were drawn from site records, including age, sex, birth origin (wild or captive), rearing condition (maternal, nursery, or other), duration of captivity and sanctuary residence, medical condition(s), dominance rank, and current social group composition.

Frequency analyses were performed to determine the prevalence of clusters of behaviors that we used as alternative criteria for PTSD and depression, and the wild and sanctuary populations were compared. A composite score was used for the purposes of the study, to determine if a chimpanzee met alternative criteria for PTSD or depression. Differences were tested for statistical significance using a Mann-Whitney U Test. An α of 0.05 was used for all statistical tests, using SPSS, version 18.0.

## Results

### Phase 1

In phase 1, a small number of chimpanzees met DSM-IV criteria for post-traumatic stress disorder or for major depression, according to raters (Table 4). For major depression, one rater deemed three chimpanzees met DSM-IV criteria. However, the other two raters found no chimpanzees who met DSM-IV criteria for major depression. For PTSD, using the DSM-IV criteria, one rater deemed that four chimpanzees met criteria; another rater deemed that two chimpanzees met criteria; and another rater deemed that three chimpanzees met criteria.

**Table 4:** Number of cases in Phase 1 diagnosed with post-traumatic stress disorder (PTSD) and major depression (MDD) using two different sets of criteria.

PTSD	Rater	DSM-IV Criteria	Alternative Criteria
	1	4/20	3/20
	2	2/20	3/20
	3	3/20	3/20
	Average number of cases diagnosed per rater	3 of 20	3 of 20

MDD	Rater	DSM-IV Criteria	Alternative Criteria
	1	3/20	0/20
	2	0/20	2/20
	3	0/20	3/20
	Average number of cases diagnosed per rater	1 of 20	1.6 of 20

We used a selection process to test different sets of alternative criteria for PTSD and depression for use in the remainder of the study, by considering items' face validity and reliability coefficients, the frequency with which items were reported in the case studies, and consultation with experts. Using the optimal set of alternative criteria for PTSD we identified, three raters agreed the same three chimpanzees met diagnostic criteria. Using the optimal set of alternative criteria for depression we identified, one rater deemed that three chimpanzees met criteria; another rater deemed two chimpanzees met criteria; and another rater deemed that no chimpanzees met criteria.

Measures of reliability were higher using the alternative criteria for PTSD and depression, compared with the DSM-IV criteria for these disorders. For PTSD, percentage agreement among the three raters for each of the 21 items for the DSM-IV criteria ranged from 27% to 73% (median = 43%), while the inter-rater reliability Κ values for the three raters ranged from −0.30 to 0.30 (median = 0). In contrast, percentage agreement among the three raters for each of the items in the alternative criteria for PTSD ranged from 63% to 100% (median = 82%), and inter-rater reliability Κ values ranged from 0.10 to 0.70 (median = 0.50). The percentage agreement among the three raters for each of the 13 DSM-IV items for major depression ranged from 12% to 67% (median = 48%), and the inter-rater reliability Κ values for the three raters ranged from −0.40 to 0.40 (median Κ = −0.10). In contrast, percentage agreement among the three raters for each of the items in the alternative criteria for depression ranged from 63% to 100% (median = 87%) and inter-rater reliability Κ values ranged from 0.10 to 1.0 (median = 0.50).

### Phase 2

**Figure 1:**
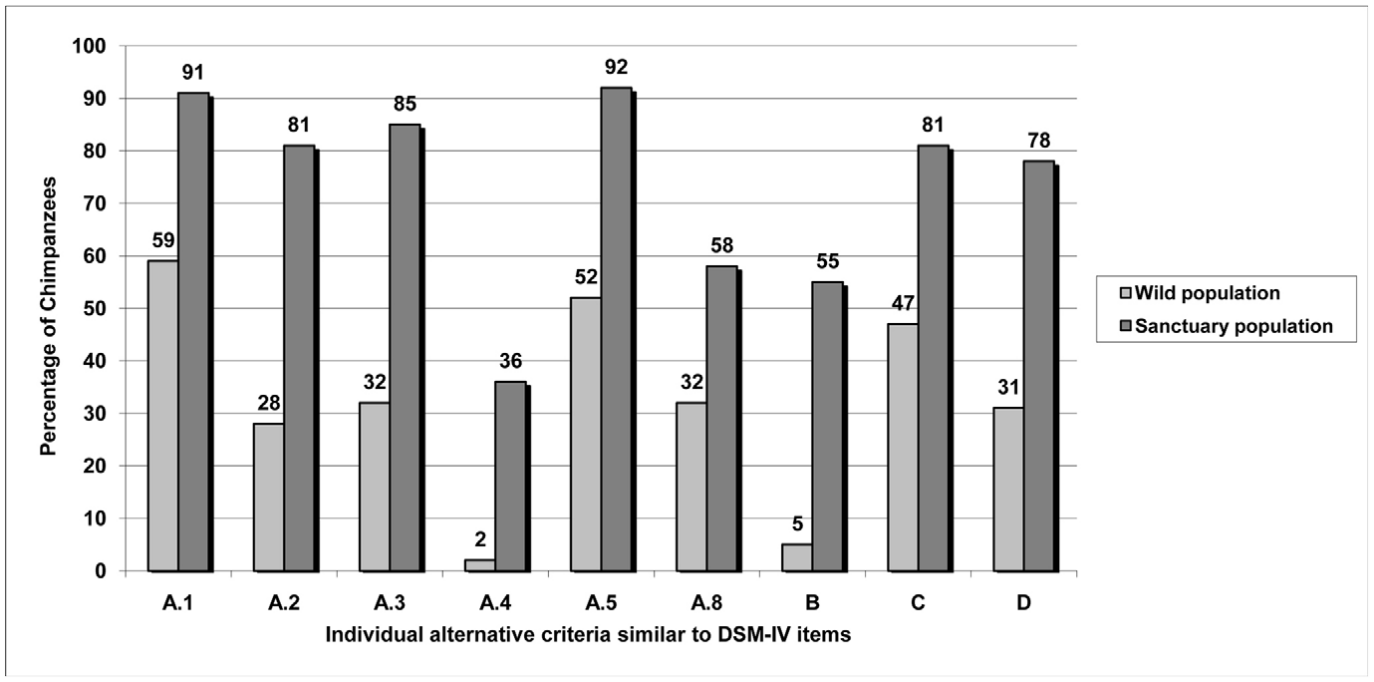
Percentage of chimpanzees displaying proposed individual alternative criteria for depression in Phase 2.

**Figure 2:**
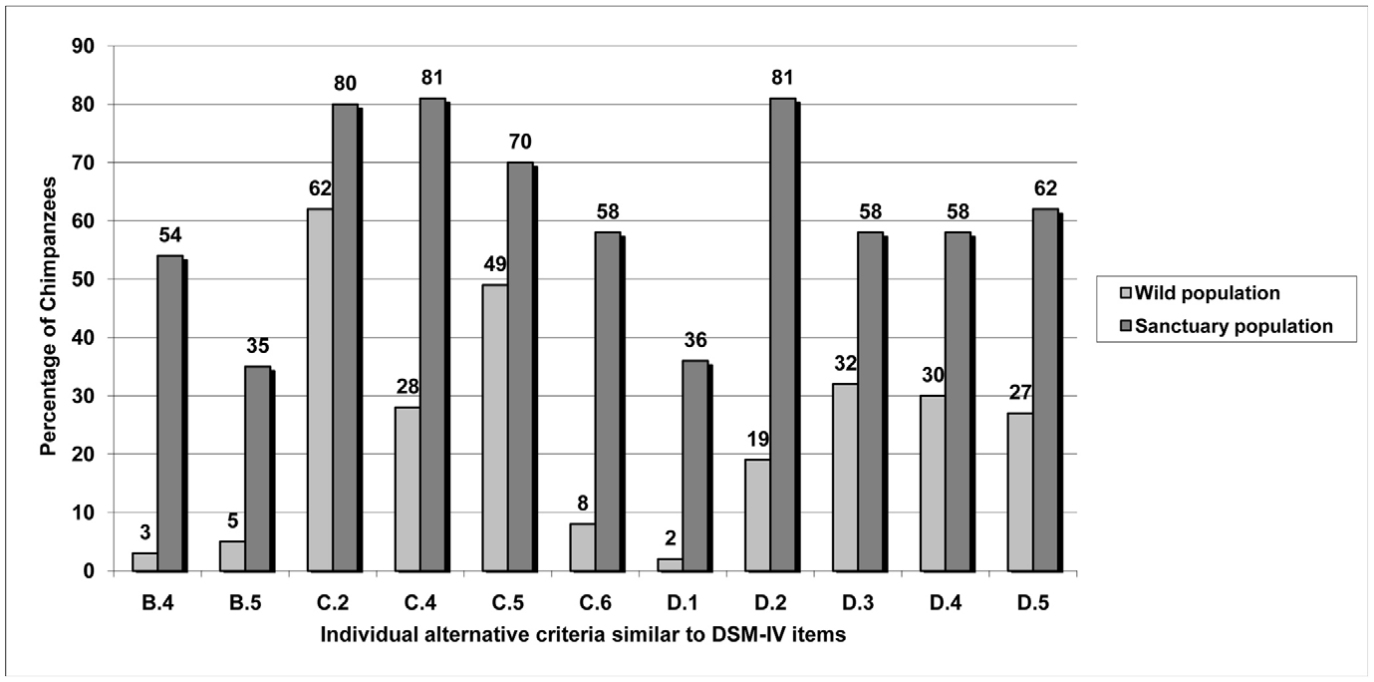
Percentage of chimpanzees displaying proposed individual alternative criteria for PTSD in Phase 2.

**Figure 3:**
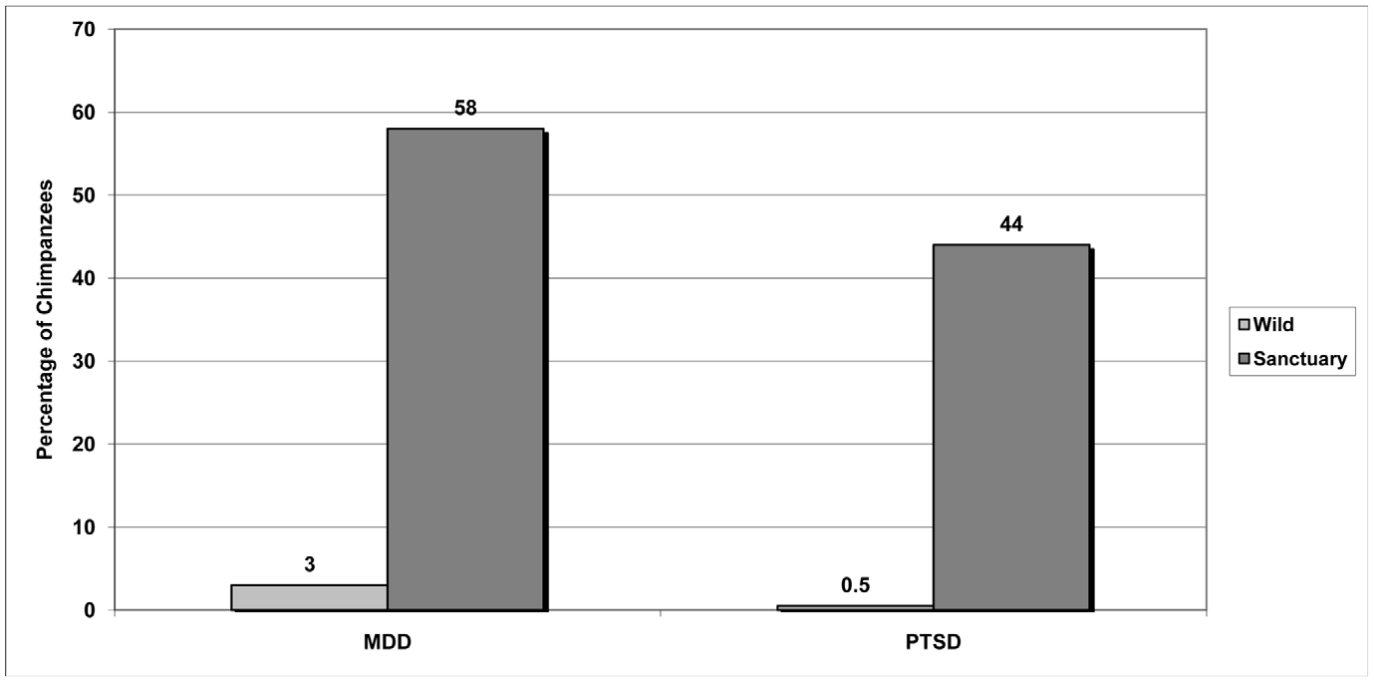
Prevalence of psychiatric disorders among chimpanzees living in the wild and in sanctuaries (Phase 2).

In phase 2, there were differences between chimpanzees living in sanctuaries and those living in the wild in the percentages of abnormal behaviors (alternative criteria developed in phase 1) reported, as shown in Figures 1 and 2. When we tested the clusters of alternative criteria (i.e. the sets of criteria shown in Tables 2 and 3), there were significant differences between chimpanzees living in sanctuaries, compared with chimpanzees living in the wild. As shown in Figure 3, 58% of chimpanzees living in sanctuaries met the definition for depression based on an alternative set of criteria, compared with 3% of chimpanzees living in the wild (p = 0.04). Similarly, 44% of chimpanzees in sanctuaries met the definition for PTSD based on alternative criteria, compared with 0.5% of chimpanzees in the wild (p = 0.04). Where duration of behavior was reported, approximately 89% of the individual items for alternative criteria for depression (Figure 1) and PTSD (Figure 2) were reported to persist for at least 1 year. Upon retest, percentage agreement within raters for individual symptoms averaged from 81% to 97%, with Cohen's kappa averaging from 0.40 to 0.80 at the eight sites.

### Case Vignettes

#### Negra

A chimpanzee named Negra was a 36-year-old female at the time of the study. Taken from the wild in Africa as an infant, she has remained in captivity since that time. She was used in invasive research, including hepatitis experiments, and for breeding. Each of her infants was removed from her at an early age. During the period in which she was used in research, she was kept in isolation for several years. Approximately 1 year prior to the study, she was transferred to Chimpanzee Sanctuary Northwest in Washington state, where she currently lives with six other chimpanzees.

Negra met alternative criteria for depression and PTSD. According to reports, she had persistent depressed hunched posture, and she was socially withdrawn. Negra slept excessively during the daytime, and she lacked interest in play, food, other individuals, and grooming. She also demonstrated poor attention to tasks. She was described as slow and sluggish, and at times, she appeared anxious. In response to an unexpected touch, she would “threat bark,” scream, or run away. Compared with other chimpanzees, she demonstrated less variability in her facial expressions. Caretakers reported that her face was expressionless, “like a ghost,” for at least a month after she arrived at the sanctuary. She seldom, if ever, exhibited a play face. She was tested for a thyroid disorder and assessed for other medical causes of her clinical presentation, but all laboratory tests were within normal limits. Based on later reports provided by her caretakers, some of her symptoms have improved since she has been living in the sanctuary. She has become more interested in other chimpanzees, including grooming, and the variability in her facial expressions has increased.

#### Mawa

A chimpanzee named Mawa was a 14-year-old male at the time of the study. He arrived at the Ngamba Island sanctuary in Uganda at approximately 3 years of age. Mawa originated from Congo, where he was kept as a family pet for about one year before being confiscated. He had serious injuries from a rope tied around his waist when he arrived at the sanctuary, but these wounds healed normally and have not caused any lasting disability.

Mawa met alternative criteria for depression and PTSD. Once at the sanctuary, Mawa initially resided with a group of younger chimpanzees and was designated as an alpha male, according to sanctuary staff. His group was later integrated into a larger mixed-age group. Mawa did not adjust quickly to the change in his social milieu, including his lower relative rank once he was living with adults. This led to conflict and antagonism with large, adult males after the integration. When fleeing fights, he would sometimes escape over an electrified fence. To mitigate the conflict, Mawa was moved to temporary housing in an enclosure adjacent to another adolescent and two juveniles. Despite efforts for reintegration, Mawa did not cope successfully and thus remained in the transitional housing for some time, extending through the study period.

Survey responses revealed that Mawa showed a number of avoidance and fear behaviors. Signs of hyperarousal, such as excessive displaying and being easily irritated or angered, were also reported. Despite limited social companionship during this time, Mawa did not show significant interest in grooming chimpanzees in adjacent enclosures. He was reported to engage in some self-directed behaviors when alone, as well as feces smearing.

In spite of his challenges, Mawa interacts with caregivers, including receiving tickling and grooming from them. Following the study period, Mawa started a new reintegration process with the larger chimpanzee group with some success. He has made progress in integration and some of the signs reported during the study have diminished. He shows some positive, pro-social behaviors.

#### Eslom

A chimpanzee known as Eslom was born in 1994 in a wild chimpanzee group that has been observed routinely for a scientific research and conservation program in Uganda. At the time of the study, Eslom was 16 years of age, and he remains a member of the same group. His history indicated that Eslom's mother died in 2007. Since that time, he has provided some caregiving for his younger sister, who exhibited ongoing behavioral effects from being orphaned at a young age. During the time of the study, Eslom's sister, Euro, disappeared from the group unexpectedly. According to reports, Eslom showed signs of “missing” Euro.

Like several other members of his community, records showed that Eslom has a history of having been caught in a snare. His left arm was ensnared in June 2009, but he was able to pull the snare from the ground and drag the snare along with him. Because the injury was substantial, a veterinary team tracked Eslom through the forest and tried to shoot him with a tranquilizer gun to remove the snare and provide veterinary care. The veterinary team was not successful as Eslom kept avoiding them. Eslom spent a significant time away from the main groups followed by the observers, and his whereabouts were unknown by observers for approximately two months. After two months, Eslom reappeared in the community without the snare. His wounds healed normally and there were no lasting, physical disabilities stemming from the snare injury.

Raters reported that Eslom exhibited several ongoing behaviors consistent with the alternative criteria for PTSD. Some behaviors had an onset of two to five years before the survey, although some behaviors were first observed within the last year. Raters reported that Eslom has shown a particular fear of humans since the summer of 2009 and that he retreated when cameras were pointed at him. Results reflected that Eslom exhibits several behaviors associated with hyperarousal, including excessive displaying and screaming, and being easily irritated or angered. It was noted that his screaming behavior changed with the change in his group status. He remained aroused for unusually long periods of time, and it took him a long time for him to calm down after incidents. Observers described that these episodes were suggestive of “tantrums”. Avoidance and fear behaviors were reported, along with social withdrawal. Eslom was often the first to awaken and leave sleeping nests in the morning. Unlike most male chimpanzees of his age, Eslom did not engage in male-male grooming. He spent a significant amount of time grooming with his sister, more than any other chimpanzee. Eslom rarely affiliated with other group members. He traveled through the forest alone or with his sister.

Today, Eslom is more tolerant of observers and he continues to spend a significant amount of time with his sister.

## Discussion

Our study shows that previously traumatized chimpanzees demonstrate persistent abnormal objective symptoms and that these symptoms cluster into syndromes that are similar to PTSD and depression. We began this investigation with three mutually exclusive hypotheses. The first hypothesis, that a number of traumatized chimpanzees would reliably meet DSM-IV criteria for PTSD or depression, was not wholly supported in phase 1. Although a number of chimpanzees met DSM-IV criteria for PTSD and depression in this phase, measures of inter-rater reliability were low. The second hypothesis, that chimpanzees would manifest few, if any, criteria for PTSD or depression, was also not supported. In both phase 1 and phase 2, multiple signs of abnormal chimpanzee behaviors similar to the criteria for PTSD and depression were reported. The third hypothesis, that chimpanzees would display clusters of symptoms that are similar to disorders such as PTSD and depression, was supported. Measures of reliability were greater among raters when testing the alternative criteria. This is likely partly attributable to attempts to reduce the need to make inferences about thoughts or feelings.

The significant differences we found in phase 2 between the wild and sanctuary populations in the percentage of chimpanzees who met a set of alternative diagnostic criteria for PTSD and depression suggest that syndromes similar to PTSD and depression are identifiable in previously traumatized chimpanzees. We propose that these diagnostic terms be used, when applicable, in chimpanzees and offer criteria for their use.

The study has important limitations. Chimpanzees cannot clearly articulate their thoughts, feelings, and experiences to humans, although a few have learned to communicate using sign language. In phase 1, ratings of psychopathology were made from published studies and case reports. These reports may have contained biases that could have influenced the raters. It is also possible that these reports did not include sufficient information for raters to accurately assess the cases for evidence of mood and anxiety disorders. The low measures of inter-rater reliability observed for some of the items composing the alternative criteria for depression and PTSD merits further exploration. However, the optimal sets of alternative criteria for PTSD and depression were tested and selected based on multiple factors, including the frequency with which items were reported in the case studies, each items' face validity and reliability coefficients, and consultation with experts. It is likely that reliability would have improved further with more training of raters. It was reassuring that there was 100 percent agreement (K = 1.0) among raters for the diagnosis of PTSD, when testing the set of alternative criteria in phase 1. Since there is no gold standard for the assessment of mood and anxiety disorders in chimpanzees, it is not possible to establish the external validity of the alternative criteria we developed.

In phase 2, the design was not longitudinal and therefore did not allow for full assessment of the duration of symptoms and impeded the assessment of severity. The study was not designed to capture simultaneous observations, therefore making it difficult to assess inter-rater reliability. Observers were asked to recall specific behaviors over differing time periods (the time in which they knew the chimpanzees). A question might arise about the comparability of data collection in the wild and sanctuary chimpanzees. Therefore, it is important to note that chimpanzees at the wild sites are typically observed for five days per week from dawn to dusk as part of systematic behavioral study. The raters at the sites have job duties which require them to observe and record very detailed features of behavior on a daily basis. The chimpanzees are recognized by unique features, such as scars, freckles, and other facial and body features that require clear view. When animals are not in close physical proximity, raters from the wild sites use high-quality binoculars to view them to ensure proper individual identification and detailed behavioral data. In our review of ethograms, we noted that some of the wild chimpanzee behaviors recorded included facial expressions and fine motor tasks. Additionally, chimpanzees at some of the sanctuaries can roam freely outdoors and out of view of the raters, sometimes comparable to distances from raters in the wild. However, given the extensive work that the raters have with the chimpanzees, there were sufficient opportunities to view the detailed study behaviors in wild and captive settings. It is likely that the prevalence of behaviors such as physiological reactions (e.g. heavy or irregular breathing) would be underestimated, rather than overestimated, at the wild and sanctuary sites.

Possible refinements for future studies include greater attention to behavioral duration and severity, respondent experience, and incorporation of other abnormal behaviors specific to chimpanzees, such as self-mutilation, smearing of feces, urophagia, and regurgitation and reingestion. In terms of the present study, it is important to note that the approach taken does not account for disorders that may occur in chimpanzees but not in humans, or for the assessment of more global dysfunction that may be measured using instruments such as surveys on subjective well-being [54], among others. Symptoms of mood and anxiety disorders can be disabling, even when full diagnostic criteria for specific psychiatric disorders are not met, as is often the case in human children and adolescents [71].

Strengths of the current study include a robust sample size in phase 2 representing a wide age range of both sexes, as well as reports of trained observers, the broad spectrum of behaviors assessed, and the ability for caregivers to report observations in a relatively unrestricted but structured setting. The clusters of symptoms, as revised, had face validity, in that they were similar to symptoms for PTSD and depression listed in the DSM-IV.

Future research may include further tests for validity and reliability, along with the evaluation of correlates for specific items and diagnoses using the alternative criteria described in our study. Additionally, longitudinal follow-up would be helpful for the interpretation of this study's findings.

Our study's findings underscore the association between psychopathology and conditions that include captivity, confinement, physical harm, loss of social bonds, and isolation. Mood and anxiety disorders such as PTSD and depression are commonly diagnosed among humans exposed to significant acute, recurrent, or persistent trauma [72]–[76]. This study suggests that chimpanzees can exhibit syndromes similar to PTSD and depression as a result of potentially traumatic experiences.

## References

[1] Brent L, Lee DR, Eichberg JW (1989). The effects of single caging on chimpanzee
behavior.. Lab Anim Sci.

[2] Capitanio JP, Erwin MJ (1986). Behavioral pathology.. Comparative primate biology, volume 2, part A. Behavior, conservation,
and ecology.

[3] Davenport RK, Rogers CM, Bourne GH (1970). Differential rearing of the chimpanzee.. The chimpanzee, volume 3: Immunology, infection, hormones, anatomy, and
behavior.

[4] Goodall J (1986). The chimpanzees of the Gombe: Patterns of behavior.

[5] Harlow HF, Dodsworth RO, Harlow MK (1965). Total social isolation in monkeys.. Proc Natl Acad Sci U S A.

[6] Nash LT, Fritz J, Alford PA, Brent L (1999). Variables influencing the origins of diverse abnormal behaviors
in a large sample of captive chimpanzees (Pan troglodytes).. Am J Primatol.

[7] Ridley RM, Baker HF (1982). Stereotypy in monkeys and humans.. Psychol Med.

[8] Tinklepaugh OL (1928). The self-mutilation of a male Macacus rhesus
monkey.. J Mammal.

[9] Yerkes RM (1943). Chimpanzees. A laboratory colony.

[10] Harlow H, Harlow CM (1986). From learning to love: The selected papers of H.F.
Harlow.. editor.

[11] International Primatological Society (2007). IPS international guidelines for the acquisition, care and
breeding of nonhuman primates.. http://www.internationalprimatologicalsociety.org/docs/IPS_International_Guidelines_for_the_Acquisition_Care_and_Breeding_of_Nonhuman_Primates_Second_Edition_2007.pdf.

[12] Baker K (2005). Enrichment for nonhuman primates: chimpanzees.

[13] Bellanca RU, Crockett CM (2002). Factors predicting increased incidence of abnormal behavior in
male pigtailed macaques.. Am J Primatol.

[14] Bradshaw GA, Capaldo T, Lindner L, Grow G (2008). Building an inner sanctuary: complex PTSD in
chimpanzees.. J Trauma Dissociation.

[15] Lutz C, Well A, Novak M (2003). Stereotypic and self-injurious behavior in rhesus macaques: a
survey and retrospective analysis of environment and early
experience.. Am J Primatol.

[16] Novak MA (2003). Self-injurious behavior in rhesus monkeys: new insights into its
etiology, physiology, and treatment.. Am J Primatol.

[17] Hook MA, Lambeth SP, Perlman JE, Stavisky R, Bloomsmith MA (2002). Inter-group variation in abnormal behavior in chimpanzees (Pan
troglodytes) and rhesus macaques (Macaca mulatta).. Appl Anim Behav Sci.

[18] Rommeck I, Anderson K, Heagerty A, Cameron A, McCowan B (2009). Risk factors and remediation of self-injurious and self-abuse
behavior in rhesus macaques.. J Appl Anim Welf Sci.

[19] McEwen BS (1997). Possible mechanisms for atrophy of the human
hippocampus.. Mol Psychiatry.

[20] McEwen BS (2000). Allostasis and allostatic load: implications for
neuropsychopharmacology.. Neuropsychopharmacology.

[21] Butler AB, Hodos W (2005). Comparative vertebrate neuroanatomy: Evolution and adaptation.

[22] McMillan FD (2005). Mental health and well-being in animals.

[23] Gregory NG (2004). Physiology and behavior of animal suffering.

[24] Wildman DE, Uddin M, Liu G, Grossman LI, Goodman M (2003). Implications of natural selection in shaping 99.4%
nonsynonymous DNA identity between humans and chimpanzees: enlarging genus
Homo.. Proc Natl Acad Sci U S A.

[25] Brüne M (2008). Textbook of evolutionary psychiatry: The origins of
psychopathology.

[26] Stevens A, Price J (2000). Evolutionary psychiatry: A new beginning. Second edition.

[27] Fabrega H (2004). Psychiatric conditions in an evolutionary
context.. Psychopathology.

[28] Povinelli DJ, Dunphy-Lelii S (2001). Do chimpanzees seek explanations? Preliminary comparative
investigations.. Can J Exp Psychol.

[29] Horner V, Whiten A (2005). Causal knowledge and imitation/emulation switching in chimpanzees
(Pan troglodytes) and children (Homo sapiens).. Anim Cogn.

[30] McGrew WC (1992). Material culture in chimpanzees.

[31] Whiten A, Goodall J, McGrew WC, Nishida T, Reynolds V (1999). Cultures in chimpanzees.. Nature.

[32] Parker ST, Mitchell RW, Boccia ML (2006). Self-Awareness in animals and humans: Developmental
perspectives.

[33] Matsuzawa T, Tomonaga M, Tanaka M (2006). Cognitive development in chimpanzees.

[34] Povinelli DJ, Eddy TJ (1996). Chimpanzees: joint visual attention.. Psychol Sci.

[35] Hare B, Call J, Tomaselllo M (2006). Chimpanzees deceive a human competitor by hiding.. Cognition.

[36] Tomonaga M, Matsuzawa T, Tomonaga M, Tanaka M (2006). Development of chimpanzee social cognition in the first two years
of life.. Cognitive development in chimpanzees.

[37] Inoue S, Matsuzawa T (2007). Working memory of numerals in chimpanzees.. Curr Biol.

[38] Beran MJ, Pate JL, Richardson WK, Rumbaugh DM (2000). A chimpanzee's (Pan troglodytes) long-term retention of
lexigrams.. Anim Learn Behav.

[39] Institute of Laboratory Animal Research, Commission on Life Sciences,
National Research Council (1996). Guide for the care and use of laboratory animals.

[40] Brüne M, Brüne-Cohrs U, McGrew WC, Preuschoft S (2006). Psychopathology in great apes: concepts, treatment options and
possible homologies to human psychiatric disorders.. Neurosci Biobehav Rev.

[41] Bradshaw GA, Sapolsky RM (2006). Mirror, mirror.. Am Scientist.

[42] Bradshaw GA, Schore AN (2007). How elephants are opening doors: developmental neuroethology,
attachment and social context.. Ethology.

[43] Bradshaw GA, Schore AN, Brown JL, Poole JH, Moss CJ (2007). Elephant breakdown.. Nature.

[44] Orosz SE, Bradshaw GA (2007). Avian neuroanatomy revisited: from clinical principles to avian
cognition.. Vet Clin North Am Exot Anim Pract.

[45] Overall KL (2000). Natural animal models of human psychiatric conditions: assessment
of mechanism and validity.. Prog Neuropsychopharmacol Biol Psychiatry.

[46] Koob GF, Ehlers CL, Kupfers DJ (1989). Animal models of depression.

[47] American Psychiatric Association (2000). Diagnostic and statistical manual of mental disorders, fourth edition
(text revision).

[48] Dehon C, Scheeringa MS (2006). Screening for preschool posttraumatic stress disorder with the
Child Behavior Checklist.. J Pediatr Psychol.

[49] Scheeringa MS, Zeanah CH, Drell MJ, Larrieu JA (1995). Two approaches to the diagnosis of posttraumatic stress disorder
in infancy and early childhood.. J Am Acad Child Adolesc Psychiatry.

[50] Scheeringa MS, Zeanah CH, Myers L, Putnam FW (2003). New findings on alternative criteria for PTSD in preschool
children.. J Am Acad Child Adolesc Psychiatry.

[51] Houlihan D, Rodriguez R, Levine HD, Kloeckl J (1990). Brief report: validation of a reinforcer survey for use with
geriatric patients.. Behav Interventions.

[52] Alexopoulos GS, Abrams RC, Young RC, Shamoian CA (1988). Cornell Scale for Depression in Dementia.. Biol Psychiatry.

[53] O'Connor LE, Berry JW, Landau V, King J, Pederson A, Landau V (2001). Chimpanzee psychopathology and subjective well-being and social
adjustment.. ChimpanZoo 2000 conference proceedings.

[54] King JE, Landau VI (2003). Can chimpanzee (Pan troglodytes) happiness be estimated by human
raters?. J Res Personality.

[55] Scott M, King JE (2006). Assessing the effects of private ownership on chimpanzees through
personality testing. War of the worlds: Chimpanzee protection versus
chimpanzee exploitation.

[56] King JE, Figueredo AJ (1997). The five-factor model plus dominance in chimpanzee
personality.. J Res Personality.

[57] Pederson AK, King JE, Landau VI (2003). Chimpanzee (Pan troglodytes) personality predicts
behavior.. J Res Personality.

[58] King JE, Weiss A, Sisco MM (2008). Aping humans: age and sex effects in chimpanzee (Pan troglodytes)
and human (Homo sapiens) personality.. J Comp Psychol.

[59] Lilienfeld SO, Gershon J, Duke M, Marino L, de Waal FB (1999). A preliminary investigation of the construct of psychopathic
personality (psychopathy) in chimpanzees (Pan troglotydes).. J Comp Psychol.

[60] Turner CH, Davenport RK, Rogers CM (1969). The effect of early deprivation on the social behavior of
adolescent chimpanzees.. Am J Psychiatry.

[61] Menzel EW, Davenport RK, Rogers CM (1963). The effects of environmental restriction upon the
chimpanzee's responsiveness to objects.. J Comp Physiol Psychol.

[62] Clark AS, Juno CJ, Maple TL (1982). Behavioral effects of a change in the physical environment: a
pilot study of captive chimpanzees.. Zoo Biol.

[63] Bradshaw GA, Capaldo T, Lindner L, Grow G (2009). Developmental context effects on bicultural posttrauma self
repair in chimpanzees.. Dev Psychol.

[64] Bourgeois SR, Vazquez M, Brasky K (2007). Combination therapy reduces self-injurious behavior in a
chimpanzee (Pan Troglodytes Troglodytes): a case report.. J Appl Anim Welf Sci.

[65] Howell SM, Fritz J, Downing S, Bunuel M (1997). Treating chronic regurgitation behavior: a case
study.. Lab Anim.

[66] Struck K, Videan EN, Fritz J, Murphy J (2007). Attempting to reduce regurgitation and reingestion in a captive
chimpanzee through increased feeding opportunities: a case
study.. Lab Anim (NY).

[67] Struthers EJ, Bloomsmith MA, Alford PL (1990). A case history of a decrement in maternal competence in a captive
chimpanzee (Pan troglodytes).. Brown University Lab Primate Newsletter.

[68] Pfeiffer AJ, Koebner LJ (1978). The resocialization of single-caged chimpanzees and the
establishment of an island colony.. J Med Primatol.

[69] Noon C (1991). Resocialization of a group of ex-laboratory chimpanzees, Pan
troglodytes.. J Med Primatol.

[70] Hasegawa T, Hiraiwa-Hasegawa M (1988). A case of offspring desertion by a female chimpanzee and the
behavioral changes of the abandoned offspring.. Primates.

[71] Giaconia RM, Reinherz HZ, Silverman AB, Pakiz B, Frost AK (1995). Traumas and posttraumatic stress disorder in a community
population of older adolescents.. J Am Acad Child Adolesc Psychiatry.

[72] Wilson JP, Keane TM (2004). Assessing psychological trauma and PTSD. Second edition.

[73] Kessler RC, Sonnega A, Bromet E, Hughes M, Nelson CB (1995). Posttraumatic stress disorder in the National Comorbidity
Survey.. Arch Gen Psychiatry.

[74] Brady KT, Killeen TK, Brewerton T, Lucerini S (2000). Comorbidity of psychiatric disorders and posttraumatic stress
disorder.. J Clin Psychiatry.

[75] Breslau N, Yehuda R (1998). Epidemiology of trauma and posttraumatic stress
disorder.. Psychological trauma.

[76] McQuaid JR, Pedrelli P, McCahill ME, Stein MB (2001). Reported trauma, post-traumatic stress disorder and major
depression among primary care patients.. Psychol Med.

